# Supplementary material for: Giant capsids from lattice self-assembly of cyclodextrin complexes
Source: Nat Commun. 2017 Jun 20;8:15856. doi: 10.1038/ncomms15856 (PMC5481833; doi:10.1038/ncomms15856)
Supplement: Supplementary Information — Supplementary Figures, Supplementary Discussion and Supplementary References [file ncomms15856-s1.pdf]

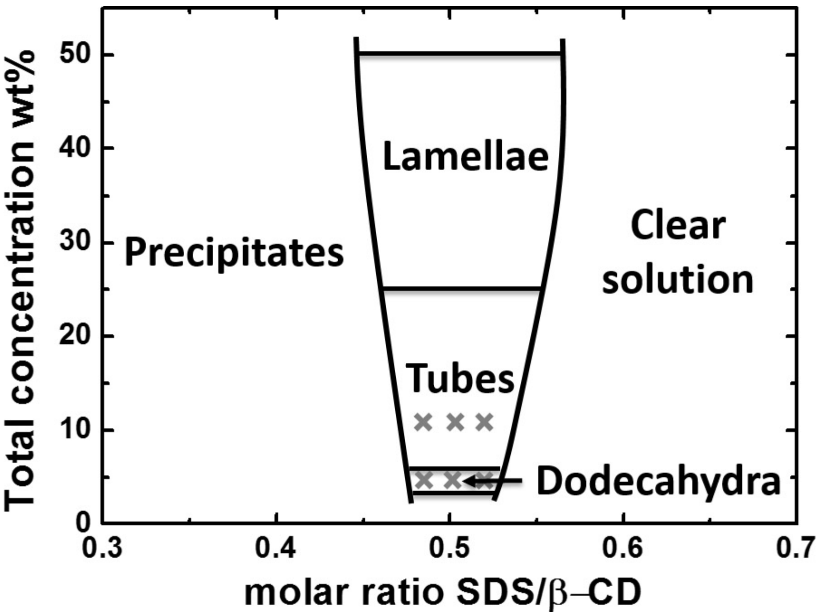

Supplementary Figure 1. The general phase diagram of the SDS/β-CD aqueous solution at room temperature. Along the SDS/β-CD ratio axis, plate crystal precipitates tend to form at low ratio because β-CD is of very limited solubility in water, and the solution is clear without any observable aggregates at high ratio because SDS/β-CD 1/1 complex is of excellent solubility in water. Near the 1/2 stoichiometry line, the complexes form lamellar, tubular, and polyhedral structures depending on the concentration. Although the current paper is focused on the assembly behavior along the stoichiometry line, we did measure the SDS/β-CD ratio inside assembly structures using PGSE-NMR (pulsed-gradient Spin-Echo nuclear magnetic resonance) method for a few selected samples that are a bit off the stoichiometry line (crosses in the phase diagram). Briefly, this method can measure the concentrations of free SDS and β-CD molecules that do not participate into the assembly structures because free molecules are of high diffusivity. For details of this method, please see our previous publication.[1] According the results, we conclude that the SDS/β-CD ratio inside the assembly structures is always very close to 1/2 (at least 98% of the complexes are in the 1/2 form) even when the bulk ratio is a bit off 1/2.

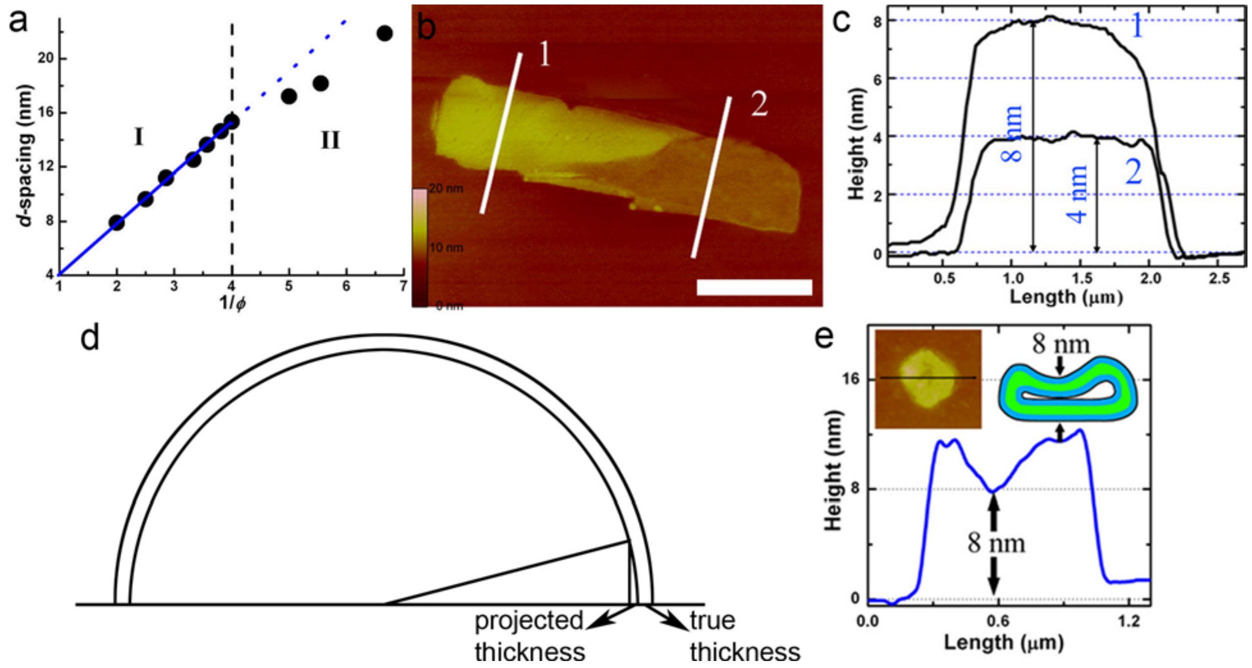

Supplementary Figure 2. The thickness of the SDS@2β-CD layer. a, The swelling behavior in lamellar (I) and tubular (II) phases. **b and c,** AFM measurements of a single-walled, collapsed tube. Scale bar: 2 μm. **d,** The apparent, projected, and true thicknesses in cryo-TEM observations. **d,** AFM measurements of a single-shell, collapsed polyhedron. The layer thickness of the lamellar, tubular, and polyhedral structures is determined to be about 4 nm by a battery of independent methods. The lamellar phase is governed by the classic swelling relationship, $d = t/\varphi$, where the repeat distance d is determined by the first harmonic peak q_1 ($d = 2\pi/q_1$), t is the thickness of the SDS@2β-CD layer, and φ is the volume fraction of SDS@2β-CD. A linear fitting of the points in Supplementary Figure 2a gives a t 3.9 nm. For the tubes, t is directly determined from AFM measurements (**b-c**) to be 4 nm. The layer thickness observed from the cryo-TEM pictures (Figure 2g-2i) ~10 nm is actually a sum of projected and true thicknesses (Figure S1d), thus largely overestimating t. The shell thickness of the polyhedral is identified to be 4 nm by AFM measurements (**e**). The fitting of the SAXS curves reaches a thickness of 4.1 nm (Figure 4b and 4c).

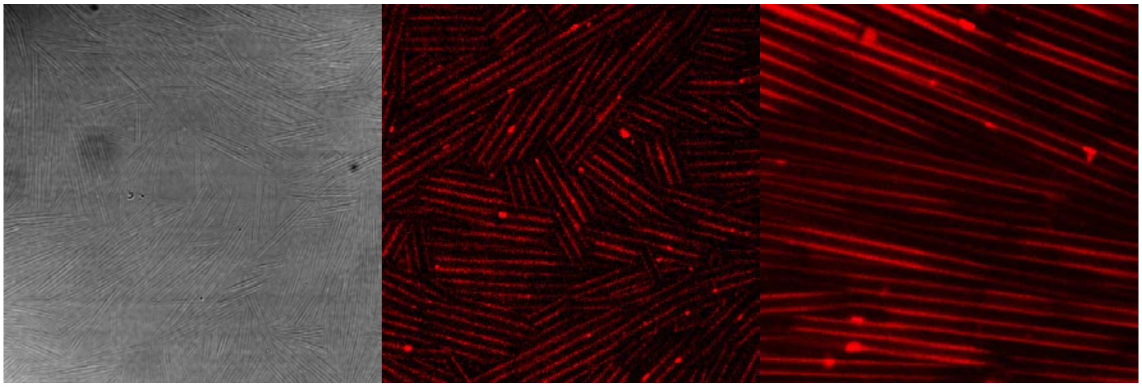

Supplementary Figure 3. Optical and Confocal Microscopy Images of SDS@2β-CD tubes for the Estimation of Persistence Length. The tube diameter is ~1.1 μm in all the three images. Since the tubes barely bend in the entire view of optical microscopy, it is very difficult to precisely measure the persistence length, *P*. We thus try to roughly estimate *P*. The average bending of the tubes (averagely ~ 40 μm long) is smaller than 1 °, so it takes 2 m long to bend 90 °. *P* is estimated to be at least on the order of 1 m, so the Young's modulus is on the order of 1 GPa.

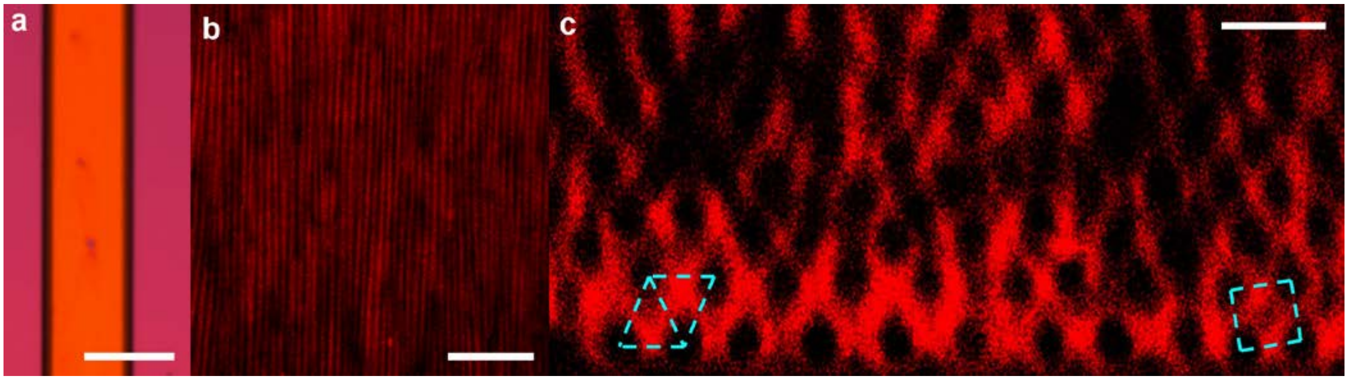

Supplementary Figure 4. Alignment of SDS@2β-CD tubes. a, The capillary loaded with aligned tubes under polarized optical microscopy, scale bar 1 mm. **b and c,** longitudinal and radial cross-sectional views of the tubes under CLSM, scale bars 15 and 3 μm, respectively. The alignment is confirmed by the uniform color in the birefringence image (**a**) and the longitudinal and radial cross-sectional views of the tubes (**b** and **c**).

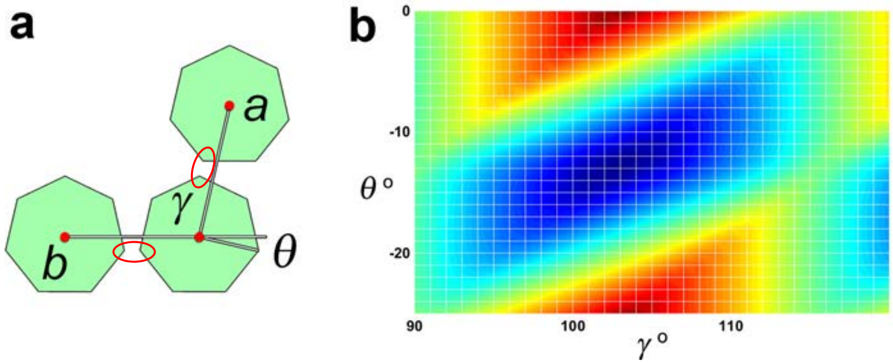

Supplementary Figure 5. The magic angel 104 ° as a consequence of maximization of the direct H-bonds with the given 7-fold molecular symmetry of β-CD. This angel appears in the nanosheets (Figure 2a-2c), precipitated flakes (Figure 2d), and the tube diffraction (Figure 4f). Here we try to use simple model to account for the angel. β-CD is a ring of 7 saccharide moieties with H-bonding sites on the rim, we thus treat it as a regular heptagon with H-bonding sites on the 7 vertices. The heptagons are arranged in a rhombic lattice, where a and b ($a = b$) are constants, γ is a variant, and the self-orientation of each heptagon θ is the other variant (**a**). It is reasonable to assume that the β-CD molecules would arrange themselves to maximize direct H-bonding by minimize the distances between H-bonding sites. The sum of the distances between two nearest H-bonding sites (highlighted by the two circles) is a function of γ and θ, which is plotted as a color map (**b**). Clearly, the minimum (dark blue) corresponds to γ = 104 °. Therefore, we argue that the γ 104 ° is a consequence of maximization of the direct H-bonds with the given 7-fold molecular symmetry of β-CD. Following this argument, we expect the lattice of α-CD and γ-CD to be hexagonal and square, respectively. In addition, we note that the exposed edges of nanosheets and flake crystals are predominantly (10) and (01) edges as a result of maximizing the direct, inter-CD H-bonding.

Supplementary Discussion

The current dodecahedra were formed by CD complexes (the building units) at a desired concentration via reversible self-assembly: at high temperature ~ 60 °C the building units are fully dissolved in water, forming no dodecahedra nor planar lattice, at room temperature the building units assemble into dodecahedra (up to 1 μm) in coexistence with a minority of planar structures (a few hundreds of nm). There are three possible pathways of dodecahedron formation: the building units add in one by one to form the dodecahedra, small planar structures (several hundreds of nm, like what we observed) assemble into the dodecahedra, and a single large planar structure (has to be several μm large) buckles into a single rhombic dodecahedron. As we did not observe any planar structure that large, we speculate that the buckling pathway is of lower possibility and that the former two pathways or their combination are of higher possibility.

It is pleasing to see the present self-assembly of hollow rhombic dodecahedra as a manifestation of the quasi-equivalence principle—the association of identical building units into highly symmetric structures due to strong intermolecular H-bonding with high efficiency and no templates[2]—although the current polyhedron size and free energy associated to pore formation might exceed the valid range of the principle. Following Lidmar et al.'s arguments,[3] the Foppel–von Karman number is defined as $\gamma = E^{2D}R^2/\kappa$, where E^{2D} is the 2D Young's modulus, R the facet size, and κ the bending rigidity. Larger γ favours buckled facets over spherical geometry. In our case, the 3D Young's modulus is estimated to be on the order of 1 GPa, so $E^{2D} \approx 4$ Pa*m and $\kappa \approx$ 6E-18 J. Given $R \approx$ 1E-6 m, the γ is on the order of 1E6. Such a large value indicates that the quasi-equivalence principle is not relevant to the formation of these shapes. It is also surprising that, unlike the icosahedral geometry favoured by catanionic surfactants and many capsid proteins, the rhombic dodecahedral geometry is dominant possibly as a consequence of the in-plane rhombic lattice.

Supplementary References

1. Jiang, L., Yu, C., Deng, M., Jin, C., Wang, Y., Yan, Y. & Huang, J. Selectivity and Stoichiometry Boosting of β-Cyclodextrin in Cationic/Anionic Surfactant Systems: When Host–Guest Equilibrium Meets Biased Aggregation Equilibrium *J. Phys. Chem. B* **114**, 2165–2174 (2010).
2. Klug, A. & Caspar, D. L. D. The structure of small viruses. *Adv. Vir. Res.* **7**, 225–325 (1960).
3. Lidmar, J ., Mirny, L. & Nelson, D. R. *Phys. Rev. E* **68,** 51910–51920 (2003).
